# Bibliometric analysis of artificial intelligence applications in cardiovascular imaging: trends, impact, and emerging research areas

**DOI:** 10.1097/MS9.0000000000003080

**Published:** 2025-02-28

**Authors:** Abdulhadi Alotaibi, Rafael Contreras, Nisarg Thakker, Abinash Mahapatro, Saisree Reddy Adla Jala, Elan Mohanty, Pavan Devulapally, Mohit Mirchandani, Mohammed Dheyaa Marsool Marsool, Shika M Jain, Farahnaz Joukar, Azin Alizadehasl, Seyedeh Fatemeh Hosseini Jebelli, Ehsan Amini-Salehi, Daniyal Ameen

**Affiliations:** a Department of Medicine and Surgery, Vision Colleges, Riyadh, Saudi Arabia; b Department of Internal medicine, Yale New Haven Health Bridgeport Hospital, Bridgeport, Connecticut; c Hi-Tech Medical College and Hospital, Rourkela, Odisha, India; d Mission Hospital, Asheville, North Carolina; e Mary Medical Center Apple Valley, California; f Main Methodist Hospital, San Antonio, Texas; g Montefiore Medical Center, Wakefield Campus, New York State; h Mayo Clinic, Scottsdale, Phoenix, Arizona; i MVJ Medical College and Research Hospital, Bengaluru, India; j Gastrointestinal and Liver Diseases Research Center, Guilan University of Medical Sciences, Rasht, Iran; k Rajaie Cardiovascular Medical and Research Center, Iran University of Medical Sciences, Tehran, Iran

**Keywords:** artificial intelligence, bibliometric analysis, cardiac imaging, cardiovascular diseases, deep learning, machine learning

## Abstract

**Background:**

The application of artificial intelligence (AI) in cardiac imaging has rapidly evolved, offering enhanced accuracy and efficiency in the diagnosis and management of cardiovascular diseases. This bibliometric study aimed to evaluate research trends, impact, and scholarly output in this expanding field.

**Methods:**

A systematic search was conducted on 14 August 2024 using the Web of Science Core Collection database. VOSviewer, CiteSpace, and Biblioshiny were utilized for data analysis.

**Results:**

The findings revealed a significant increase in publications on AI in cardiovascular imaging, particularly from 2018 to 2023, with the United States leading in research output. England and the United States have emerged as central hubs in the global research network, highlighting their role in generating high-quality and impactful publications. The University of London was identified as the top contributing institution, while *Frontiers in Cardiovascular Medicine* was the most prolific journal. Keyword analysis highlighted machine learning, echocardiography, and diagnosis as the most frequently occurring terms. A time trend analysis showed a shift in research focus toward AI applications in cardiac computed tomography (CT) and magnetic resonance imaging (MRI), with recent keywords like ejection fraction, risk, and heart failure reflecting emerging areas of interest.

**Conclusion:**

Healthcare providers should consider integrating AI tools into cardiovascular imaging practice, as AI has demonstrated the potential to enhance diagnostic accuracy and improve patient outcomes. This study highlights the rising importance of AI in personalized and predictive cardiovascular care, urging healthcare providers to stay informed about these advancements to enhance clinical decision-making and patient management.

## Introduction

Artificial intelligence (AI) is increasingly recognized as a transformative technology in healthcare, particularly in the domain of cardiovascular imaging^[1–3]^. Cardiovascular imaging plays a critical role in the diagnosis, management, and treatment of cardiovascular diseases, which remain the leading cause of mortality worldwide^[4–11]^. The interpretation of cardiac imaging data, including echocardiograms, MRI, and computed tomography (CT) scans, is complex and requires a high level of expertise^[12,13]^. Traditional image analysis methods, though effective, are often time-consuming and can vary in accuracy depending on the operator’s experience. AI offers a promising solution to these challenges by improving the accuracy, efficiency, and consistency of image analysis^[14–16]^.

**HIGHLIGHTS**

- Comprehensive analysis of AI in cardiovascular imaging reveals its growing role in enhancing diagnostic accuracy and managing cardiovascular diseases.
- Research surged from 2018–2023, with emerging hotspots in cardiac CT and MRI, indicating future trends toward personalized AI-driven cardiovascular care.
- AI is poised to revolutionize cardiovascular care through automation, personalized treatment, and improved patient outcomes.

The application of AI in cardiac imaging primarily involves machine learning and deep learning techniques, which are capable of processing vast amounts of imaging data to detect patterns, segment anatomical structures, and predict clinical outcomes^[17,18]^. These AI-driven approaches have shown significant potential in automating tasks such as image segmentation, where software can delineate the boundaries of cardiac structures with high precision^[19,20]^. This capability is crucial for accurate measurement and assessment of cardiac function, which is essential for diagnosing conditions like cardiomyopathy, coronary artery disease, and valvular heart disease^[5,21–23]^.

Moreover, AI algorithms can identify subtle imaging features that may be missed by human observers, thereby improving the early detection and diagnosis of cardiac pathologies^[24]^. For example, AI models have demonstrated efficacy in detecting myocardial infarction, heart failure, and arrhythmias with high levels of accuracy^[25–27]^. The integration of AI into cardiovascular imaging workflows also has the potential to reduce clinicians’ workloads, allowing them to focus on more complex decision-making processes and patient care^[28–31]^.

As AI technology continues to evolve, its applications in cardiovascular imaging are expected to expand, leading to more personalized and precise healthcare. The ongoing development and validation of AI algorithms, along with their integration into clinical practice, will likely enhance early diagnosis and management of cardiovascular diseases, ultimately improving patient outcomes^[25,32,33]^.

Given the rapid expansion of research in this area, a bibliometric study is essential to systematically evaluate the trends, impact, and scholarly output related to the application of AI in cardiovascular imaging. Bibliometric analysis, which involves the quantitative assessment of published literature, provides valuable insights into the development of research themes, collaboration networks among researchers, and the most influential publications and authors in the field^[34,35]^. By examining publication patterns, citation metrics, and keyword co-occurrences, bibliometric studies can help identify key areas of focus within the intersection of AI and cardiovascular imaging, as well as emerging topics that may shape future research.

This study aimed to provide a comprehensive overview of the existing literature on AI in cardiovascular imaging by analyzing publication volume, the geographical distribution of research, and the evolution of scientific contributions over time. Additionally, we aimed to identify the leading journals, institutions, and researchers at the forefront of this rapidly advancing field. Understanding these patterns can provide valuable insights into how AI applications in cardiovascular imaging are being developed and disseminated, highlighting the areas with the greatest potential for clinical impact and future research opportunities.

## Methods

### Data collection

**Figure 1.**
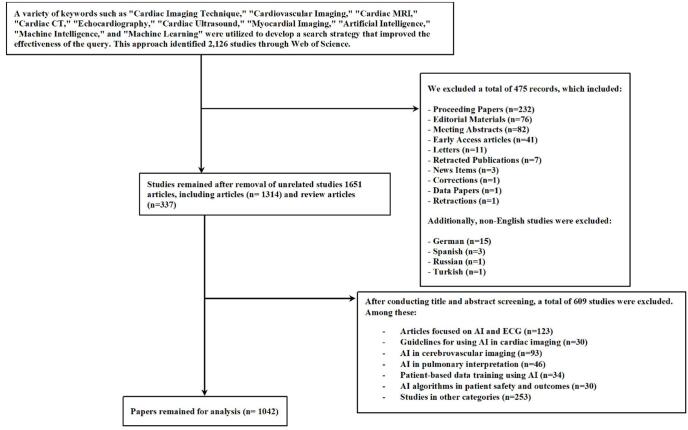
Study selection process.

To gather information on published articles, a search was conducted on 14 August 2024, using the Web of Science Core Collection, a comprehensive and credible database featuring over 12 000 reputable publications^[36–38]^. A range of keywords, including “Cardiac Imaging Technique”, “Cardiovascular Imaging”, “Cardiac MRI”, “Cardiac CT”, “Echocardiography”, “Cardiac Ultrasound”, “Myocardial Imaging”, “Artificial Intelligence”, “Machine Intelligence”, and “Machine Learning”, were employed to create a search strategy that enhanced the query’s efficacy (Table S1, http://links.lww.com/MS9/A744). Initially, 2126 papers were retrieved. After removing book chapters, editorials, conference papers, letters, and pre-publication articles, the final selection comprised 1042 publications (Fig. 1).

**Figure 2.**
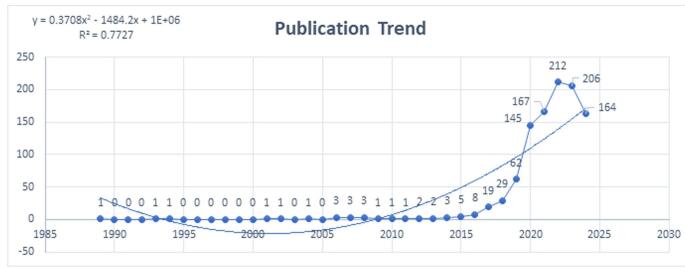
Trends in publication regarding artificial intelligence in cardiovascular imaging. (This figure illustrates the annual number of publications on the application of artificial intelligence in cardiovascular imaging over the past decades. It highlights a significant increase in research output, particularly after 2018, indicating a growing interest and emphasis in the field. The peak in publications during 2023 suggests that artificial intelligence in cardiovascular imaging is becoming a central focus for researchers.).

The decision to include only original research and review articles was based on their rigorous peer-review processes, which ensure the credibility and scientific value of the included studies. Other types of literature, such as conference proceedings, editorials, and books, were excluded because they are not typically subject to the same level of peer review or indexing, which may affect the consistency and reliability of citation trends in a bibliometric analysis.

Additionally, we excluded retracted papers to maintain the integrity of the dataset, as they may no longer reflect valid scientific contributions. Non-English publications were excluded because the bibliometric software tools we employed are optimized for processing English-language text. As these tools are not fully compatible with non-English content, limiting the dataset to English papers helped ensure consistency in the analysis.

### Data screening

To ensure the quality and relevance of the included studies, we performed a thorough screening process. We reviewed the titles, abstracts, and keywords of all retrieved publications. In cases where the relevance or quality was uncertain based on these criteria, we reviewed the full text. This process ensured that only studies related to AI applications in cardiovascular imaging were included, ensuring the focus and integrity of our analysis.

### Data analysis

VOSviewer (version 1.6.19), CiteSpace (version 6.3 R1), and Biblioshiny (version 4.0) were used to analyze the documents downloaded from the Web of Science Core Collection. The data were then converted into CSV and plain text formats.

VOSviewer (accessible at www.vosviewer.com) is a widely used tool for constructing and visualizing bibliometric networks, enabling researchers to analyze patterns in academic publications^[39]^. It is developed by Nees Jan van Eck and Ludo Waltman at the Centre for Science and Technology Studies of Leiden University^[39]^. This software specializes in visualizing networks based on co-authorship, co-citation, bibliographic coupling, and keyword co-occurrence. Co-authorship analysis reveals collaborative patterns between authors and institutions, providing insights into research collaboration trends. Co-citation analysis identifies papers or authors frequently cited together, helping to reveal intellectual connections and key research communities^[40]^. Bibliographic coupling groups documents that share common references, aiding in the identification of related topics and emerging research fronts^[41]^. Keyword co-occurrence analysis visualizes frequently occurring terms in publications, reflecting the thematic structure and trends within a field^[42]^. VOSviewer uses text mining techniques, including sentence detection and part-of-speech tagging algorithms provided by the Apache OpenNLP library. Sentence detection splits text data into individual sentences, while part-of-speech tagging assigns each word a part of speech (verb, noun, adjective), facilitating a deeper analysis of the text and allowing more accurate identification of research themes. VOSviewer also employs distance-based visualization, where the proximity of nodes represents the strength of their relationship, making it easier to interpret complex bibliometric data^[39,43,44]^.

CiteSpace, developed by Chaomei Chen at Drexel University (accessible at www.citespace.podia.com), is designed for visualizing and analyzing citation networks. It is particularly useful for identifying citation bursts and tracking emerging research trends. A citation burst indicates a rapid increase in citations of a specific paper or topic, highlighting its growing influence in the field^[45]^. CiteSpace excels at cluster analysis, which groups related studies based on co-citation patterns, revealing connections between different research areas. Clusters are labeled using log-likelihood ratio (LLR) tests, which automatically generate meaningful labels from key terms within the cluster’s articles. Cluster quality is measured by modularity and silhouette scores. Modularity assesses the internal structure of the network, with higher values (approaching 1) indicating more distinct, loosely connected sub-networks. Silhouette scores evaluate the cohesiveness of clusters, with higher scores reflecting more homogeneous and meaningful groupings. CiteSpace also incorporates time-slicing, allowing researchers to track the evolution of key concepts over specific time periods. Its burst detection algorithm, based on Kleinberg’s method, identifies sudden increases in citation activity, helping to spot emerging research trends or breakthroughs.

Biblioshiny (accessible at www.bibliometrix.org), a graphical interface for the R Bibliometrix package developed by Massimo Aria and Corrado Cuccurullo, provides comprehensive tools for bibliometric analysis, including citation analysis to measure the impact of papers, authors, and institutions, as well as co-authorship analysis to uncover collaboration networks^[39,46]^. Biblioshiny also implements various community detection algorithms, such as the Louvain, Walktrap, and Multidimensional Scaling (MDS) algorithms, which help identify clusters within networks and reveal related research areas or collaborations^[47–50]^.

## Results

### Publication trend

Trends in research within a specific field can often be understood by analyzing the number of publications produced over time. In the early stages, research on AI and cardiovascular imaging was relatively sparse, with only a few papers published between 1990 and 2017. However, from 2018 to 2023, there was a sharp increase in publications, rising from 29 papers in 2018 to 206 papers in 2023. This surge highlighted the growing interest and focus on the potential applications of AI in cardiovascular imaging. In 2024, there was a slight decline to 164 publications, likely due to the ongoing year and the typical delays in publication (Fig. 2).

**Figure 3.**
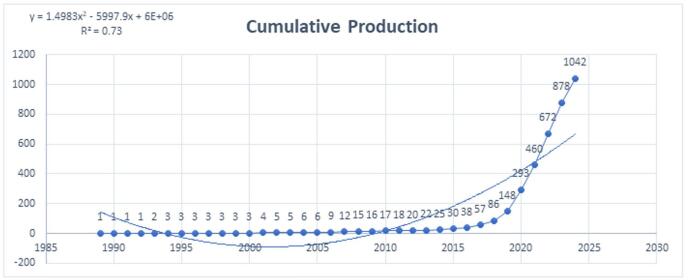
Cumulative publications regarding artificial intelligence in cardiovascular imaging. (This figure shows the cumulative growth of publications on the use of artificial intelligence in cardiovascular imaging. The data reveal a steady increase in research output from 1990 to around 2015, followed by a more rapid rise starting in 2016. This upward trend highlights the increasing recognition of artificial intelligence as a valuable tool for enhancing the accuracy, efficiency, and consistency of cardiovascular imaging.).

The cumulative research output in AI for cardiovascular imaging showed a significant upward trend over time. Initially, the growth was slow and steady, with cumulative publications remaining below 10 until 2006. Between 2006 and 2015, there was a modest increase, with the cumulative total reaching 30 papers by 2015. However, from 2016 onwards, the growth rate accelerated significantly, with the cumulative total reaching 1,042 papers by 2024 (Fig. 3). This rapid increase underscored the expanding impact and interest in AI applications within cardiovascular imaging.

### Countries and institutions

**Figure 4.**
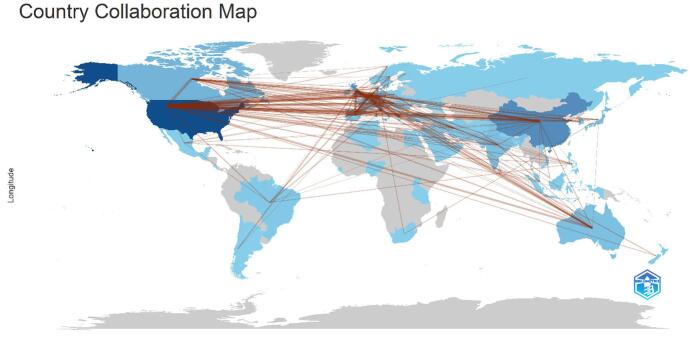
Countries’ collaboration in the field of artificial intelligence in cardiovascular imaging.

The examination of global contributions revealed that 75 countries had collaborated in this research domain (Fig. 4). The analysis of the top ten countries by publication output showed that the United States led with a substantial contribution of 421 papers, followed by China with 184 papers and England with 150 papers. Italy (*n* = 90), Germany (*n* = 88), Canada (*n* = 82), the Netherlands (*n* = 78), Japan (*n* = 58), France (*n* = 45), and Switzerland (*n* = 45) also made significant contributions to the field (Table 1).

**Table 1:** Top 10 countries by number of publications (This table illustrates the countries with the highest contributions to research on artificial intelligence in cardiovascular imaging).

Rank	Country	Number of Publications
1	USA	421
2	China	184
3	England	150
4	Italy	90
5	Germany	88
6	Canada	82
7	Netherlands	78
8	Japan	58
9	France	45
10	Switzerland	45

**Figure 5.**
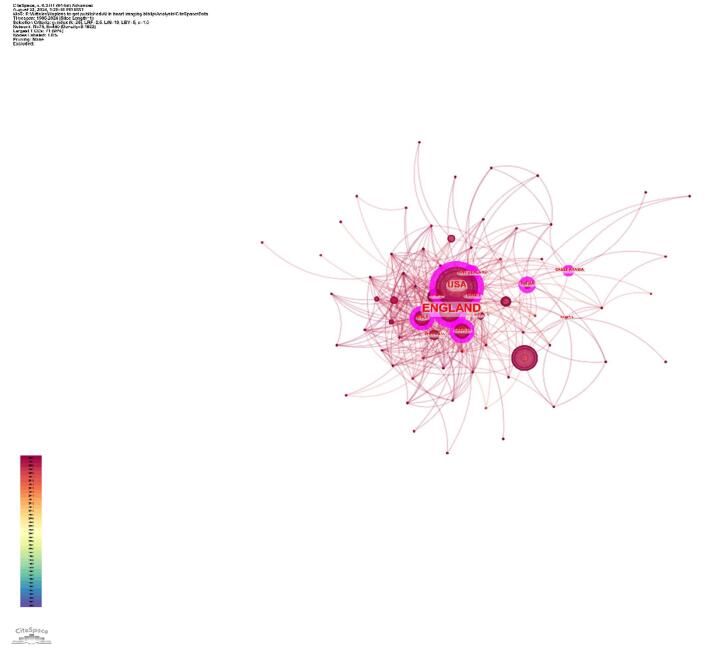
Countries with high centrality in the field of artificial intelligence in cardiovascular imaging. (This figure shows the countries with the highest impact in the global research network on artificial intelligence in cardiovascular imaging. England, the United States, and India are identified as the top three countries with the highest centrality (purple rings), indicating their key influence and leadership in advancing research and fostering global cooperation.).

**Figure 6.**
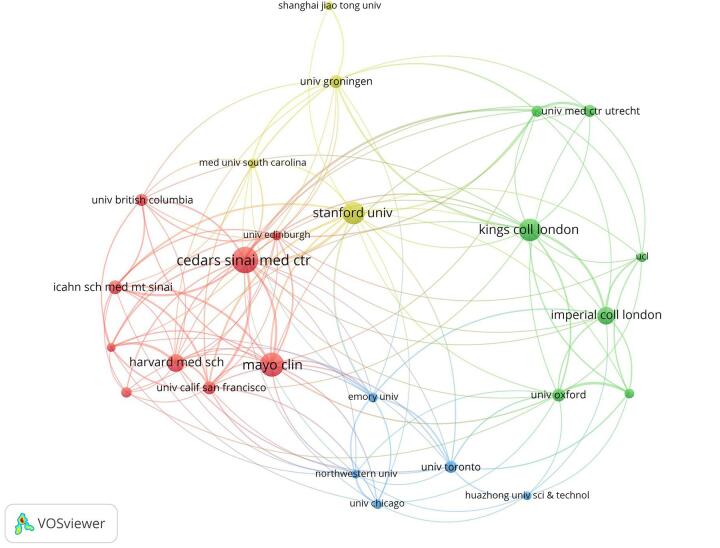
Network visualization of the countries in the field of artificial intelligence in cardiovascular imaging. (This figure depicts the collaborative relationships between different countries. A thicker line connecting two countries, along with more adjacent countries, indicates a stronger collaboration.).

The top ten countries in terms of centrality are presented in Table 2. England emerged as the country with the highest centrality (0.32), followed by the USA (0.23). Other countries with notable centrality values include India (0.16), Switzerland (0.13), Italy (0.11), and Canada (0.11). Saudi Arabia (0.11), France (0.10), Australia (0.09), and Germany (0.08) also played critical roles in maintaining the cohesion and influence of the research network (Table 2). Figure 5 illustrates the collaborative network among countries, highlighting central nodes such as England and the USA, which acted as significant centers of collaboration. Figure 6 depicts the collaboration strength among different countries.

**Table 2:** Top 10 countries by centrality (This table presents the countries with the highest centrality scores, reflecting their key roles in facilitating international collaboration and knowledge exchange in the field of artificial intelligence applied to cardiovascular imaging).

Rank	Country	Centrality
1	England	0.32
2	USA	0.23
3	India	0.16
4	Switzerland	0.13
5	Italy	0.11
6	Canada	0.11
7	Saudi Arabia	0.11
8	France	0.10
9	Australia	0.09
10	Germany	0.08

**Figure 7.**
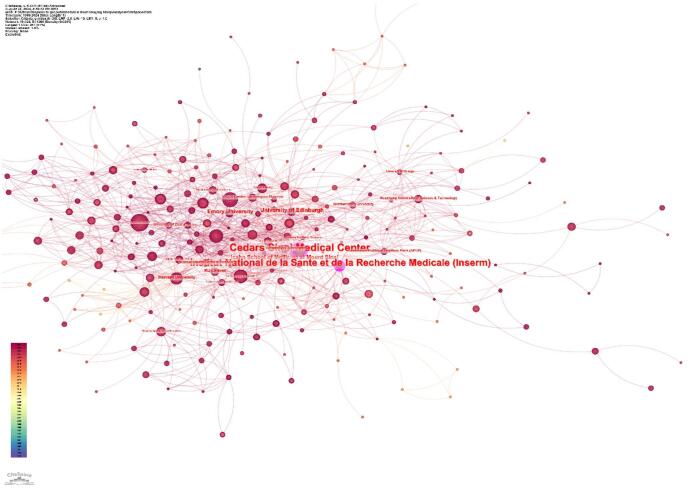
Institutions with high centrality in the field of artificial intelligence in cardiovascular imaging. (This figure highlights the institutions that play central roles in the global research network on artificial intelligence in cardiovascular imaging. Centrality indicates how influential an institution is in facilitating collaboration and knowledge exchange within the network. Cedars–Sinai Medical Center, Institut National de la Santé et de la Recherche Médicale, and Emory University are the top three institutions with the highest centrality, reflecting their critical contributions to advancing research and promoting international collaboration in this field.).

**Figure 8.**
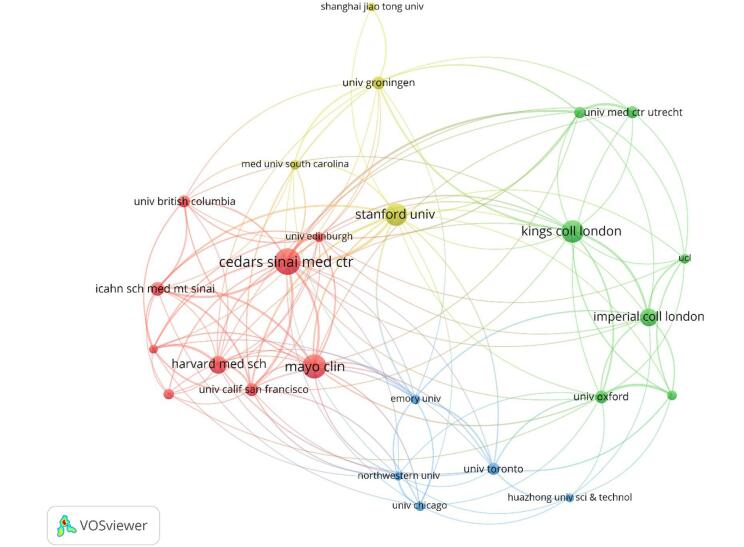
Network visualization of the institutions in the field of artificial intelligence in cardiovascular imaging. (This figure depicts the collaborative relationships between different institutions. A thicker line connecting two institutions, along with more adjacent institutions, indicates a stronger collaboration.).

Regarding institutions, the University of London led with 66 publications, followed by the University of California System with 55. Cedars-Sinai Medical Center contributed 44 publications, while Harvard University had 41. The Mayo Clinic published 40 works, with King’s College London following with 37 (Table 3 and Fig. 7). Figure 8 shows the collaboration strength among different institutions.

**Table 3:** Top 10 institutions by number of publications (This table highlights the top institutions driving research on artificial intelligence in cardiovascular imaging, showcasing their substantial contributions to advancing the field through prolific scholarly output).

Rank	Institutions	Number of Publications
1	University of London	66
2	University of California System	55
3	Cedars Sinai Medical Center	44
4	Harvard University	41
5	Mayo Clinic	40
6	King’s College London	37
7	Stanford University	33
8	Imperial College London	28
9	Harvard Medical School	26
10	Utrecht University	25

### Journals and co-cited journals

**Figure 9.**
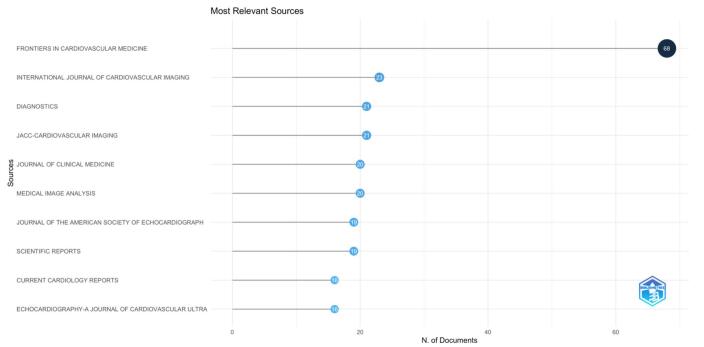
Top 10 journals in the field of artificial intelligence and cardiovascular imaging. (This figure presents the top 10 journals that have published the most research on artificial intelligence in cardiovascular imaging. Journals such as Frontiers in Cardiovascular Medicine, The International Journal of Cardiovascular Imaging, and Diagnostics lead in the number of publications in this field.).

The bibliometric analysis identified 336 journals publishing on AI in cardiovascular imaging. Frontiers in Cardiovascular Medicine led with 68 publications, followed by the International Journal of Cardiovascular Imaging with 23, and Diagnostics with 21. JACC: Cardiovascular Imaging and the Journal of Clinical Medicine contributed 21 and 20 publications, respectively. Other significant sources included Medical Image Analysis (20 publications), Scientific Reports (19 publications), Journal of the American Society of Echocardiography (19 publications), and Current Cardiology Reports (16 publications). Figure 9 illustrates the top ten journals in the field of artificial intelligence in cardiovascular imaging.

**Figure 10.**
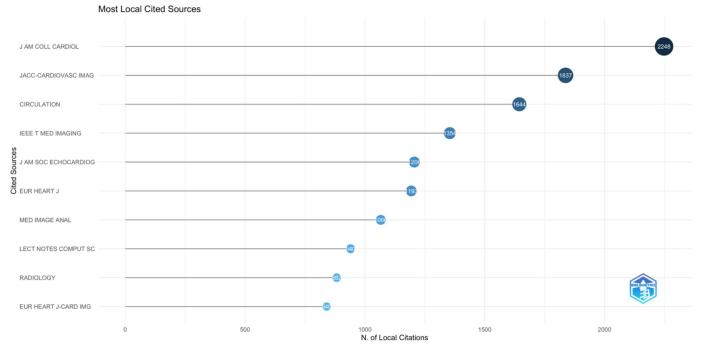
Top 10 co-cited journals in the field of artificial intelligence in cardiovascular imaging. (This figure shows the top 10 journals most frequently co-cited in research related to artificial intelligence in cardiovascular imaging. Co-citation indicates the degree to which different journals are referenced together in the same research articles, reflecting their influence on the field. The Journal of the American College of Cardiology, JACC: Cardiovascular Imaging, and Circulation are the top three co-cited journals.).

The co-citation analysis revealed that the Journal of the American College of Cardiology had the highest number of citations, with 2,248. This was followed by JACC: Cardiovascular Imaging with 1,837 citations and Circulation with 1,644 citations. IEEE Transactions on Medical Imaging received 1,354 citations, while the Journal of the American Society of Echocardiography and the European Heart Journal garnered 1,206 and 1,193 citations, respectively. Figure 10 presents the top ten co-cited journals in the field of AI in cardiovascular imaging.

**Figure 11.**
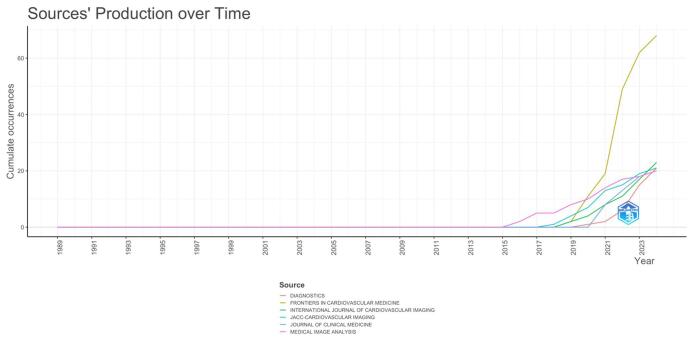
Journals’ productions over time in the field of artificial intelligence in cardiovascular imaging. (This figure tracks the publication output of leading journals in artificial intelligence research in cardiovascular imaging from 1990 to 2024. It shows a noticeable increase in the number of publications after 2015, with journals such as Frontiers in Cardiovascular Medicine experiencing the most significant rise. This growth reflects the expanding interest and development in artificial intelligence technologies for cardiovascular imaging.).

The analysis of publication trends over time, depicted in Figure 11, highlighted the evolving research output across several journals. The cumulative number of publications remained relatively low until around 2015, with minimal contributions from journals such as Medical Image Analysis. From 2016 onwards, research output increased, with journals like the Journal of Clinical Medicine and JACC: Cardiovascular Imaging starting to make notable contributions. However, the most significant growth occurred in Frontiers in Cardiovascular Medicine, which experienced a dramatic surge in publications from 2019 onwards, becoming the leading journal in the field. The overall trend showed a sharp rise in publications over the past decade, with a pronounced acceleration beginning around 2020. This reflected the growing interest and advancements in the field during this period.

**Figure 12.**
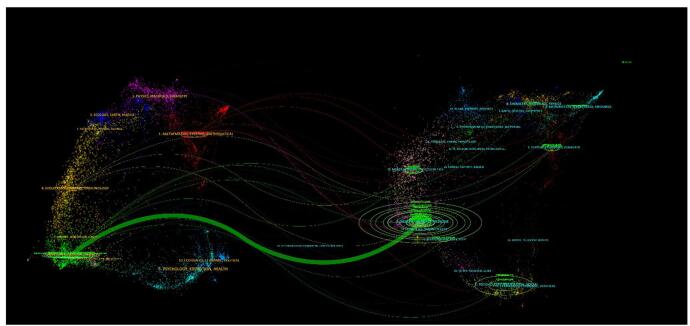
Dual over lay map of artificial intelligence in cardiovascular imaging. (This figure illustrates the dual overlay map of journal citation patterns in the field of artificial intelligence in cardiovascular imaging. The left side of the map shows the journals that are citing, while the right side shows the journals that are being cited. The overlay helps visualize the flow of information between different academic fields. The map reveals that journals in the Health, Nursing, and Medicine categories are most frequently cited by journals in the Medicine, Medical, and Clinical domains, emphasizing the interdisciplinary nature of research in artificial intelligence and cardiovascular imaging.).

A dual-map overlay, which efficiently depicted citation patterns, illustrated the relationship between citing and cited journals. As shown in Figure 12, journals in Health, Nursing, and Medicine (on the right) were frequently cited by journals in Medicine, Medical, and Clinical fields (on the left).

### Authors and co-cited authors

The analysis of contributions in the domain of AI in cardiovascular imaging highlighted several key authors who significantly influenced the research landscape. Partho P. Sengupta stood out as the leading author, with the highest number of publications at 26. Following closely, Damini Dey and Ivana Isgum each contributed 20 publications. Tim Leiner and Piotr J. Slomka also ranked highly, with 19 and 14 publications, respectively (Table 4).

**Table 4:** Top 10 authors by publications, citations, and co-citations (This table showcases the leading authors in the field of artificial intelligence for cardiovascular imaging, ranked by their number of publications, total citations, and co-citations, reflecting their significant impact and influence on the research landscape).

Number	Author with a high number of publications	Number of publications	Author with a high number of citations	Number of citations	The most Co-cited authors	Number of co-citations
1	Partho P. Sengupta	26	Ivana Isgum	1808	Roberto M. Lang	197
2	Ivana Isgum	20	Tim Leiner	1721	Partho P. Sengupta	183
3	Damini Dey	20	Jelmer M. Wolterink	1490	J. Zhang	180
4	Tim Leiner	19	Max A. Viergever	1315	Jelmer M. Wolterink	178
5	Piotr J. Slomka	14	Partho P. Sengupta	1245	O. Ronneberger	163
6	Roberto M. Lang	13	Piotr J. Slomka	1050	K. Kusunose	150
7	Steffen E. Petersen	13	Damini Dey	846	D. Dey	149
8	Kenya Kusunose	13	Daniel S. Berman	675	A. Madani	128
9	David Ouyang	12	James K. Min	658	J. Betancur	116
10	Jelmer M. Wolterink	11	Bob D. de Vos	603	G. Litjens	102

Ivana Isgum emerged as the most cited author with 1808 citations, followed by Tim Leiner with 1721 citations. Jelmer M. Wolterink also made a significant impact with 1490 citations. Max A. Viergever and Partho P. Sengupta rounded out the top five, with 1315 and 1245 citations, respectively (Table 4).

The analysis of co-citations in the field revealed that Roberto M. Lang was the most cited author, with 197 citations, followed by Partho P. Sengupta with 183 citations. J. Zhang and Jelmer M. Wolterink were also highly cited, with 180 and 178 citations, respectively. O. Ronneberger completed the top five with 163 citations (Table 4).

### Top cited papers

**Figure 13.**
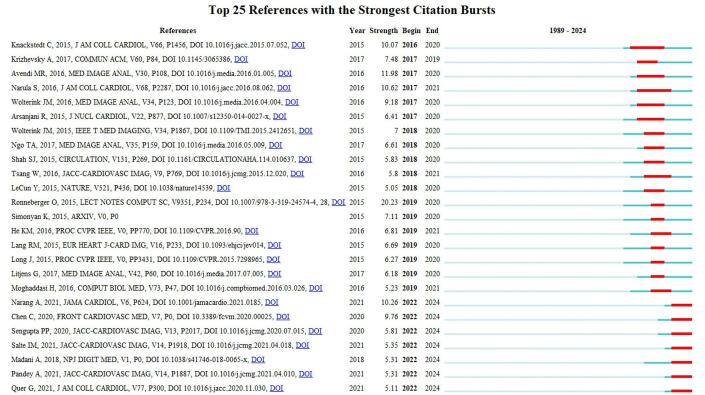
Citation burst of the papers in the field of artificial intelligence in cardiovascular imaging. (This figure displays the citation burst analysis of key papers in the field of artificial intelligence in cardiovascular imaging, identifying articles that have experienced a rapid surge in citations over a short period. Citation bursts indicate the growing relevance of certain papers in the field. The figure reveals that papers published between 2016 and 2019 have experienced strong bursts, reflecting their significant impact on subsequent research. Notably, the citation burst continues for papers published as recently as 2022, highlighting that the field is still evolving rapidly, with new influential studies emerging.).

Table 5 lists the top 10 most cited papers in the field of artificial intelligence applications in cardiovascular imaging, highlighting their significant impact on the field. The most cited paper was titled “Generative adversarial networks for noise reduction in low-dose CT”, published in 2017, with 664 citations. Following this, the 2018 article “Fully automated echocardiogram interpretation in clinical practice: feasibility and diagnostic accuracy” garnered 466 citations. The third most cited work was “A combined deep-learning and deformable-model approach to fully automatic segmentation of the left ventricle in cardiac MRI,” published in 2016, with 433 citations. The 2019 paper “Artificial Intelligence in Cardiovascular Imaging” ranked fourth with 319 citations. The fifth most cited article was “Deep learning for segmentation using an open large-scale dataset in 2D echocardiography,” also published in 2019, with 284 citations. Figure 13 illustrates the citation burst of published papers in the field. A total of 25 papers experienced a citation burst beginning in 2016.

**Table 5:** Top ten cited papers in the field of artificial intelligence in cardiovascular imaging (This table highlights the most cited papers in the field of artificial intelligence applied to cardiovascular imaging, reflecting their significant impact on shaping current research).

Number	Title of the most cited paper	Published year	Number of citations
1	Generative adversarial networks for noise reduction in low-dose CT^[51]^	2017	664
2	Fully automated echocardiogram interpretation in clinical practice: feasibility and diagnostic accuracy^[46]^	2018	466
3	A combined deep-learning and deformable-model approach to fully automatic segmentation of the left ventricle in cardiac MRI^[52]^	2016	433
4	Artificial Intelligence in Cardiovascular Imaging^[17]^	2019	319
5	Deep learning for segmentation using an open large-scale dataset in 2D echocardiography^[53]^	2019	284
6	Fast and accurate view classification of echocardiograms using deep learning^[54]^	2018	284
7	Clinical applications of machine learning in cardiovascular disease and its relevance to cardiac imaging^[55]^	2019	263
8	Artificial Intelligence in Medicine and Cardiac Imaging: Harnessing Big Data and Advanced Computing to Provide Personalized Medical Diagnosis and Treatment^[56]^	2014	238
9	Scan-specific robust artificial-neural-networks for k-space interpolation (RAKI) reconstruction: Database-free deep learning for fast imaging^[57]^	2019	237
10	Machine-Learning Algorithms to Automate Morphological and Functional Assessments in 2D Echocardiography^[58]^	2016	230

### Keyword trends, hotspots, and cluster analysis

**Figure 14.**
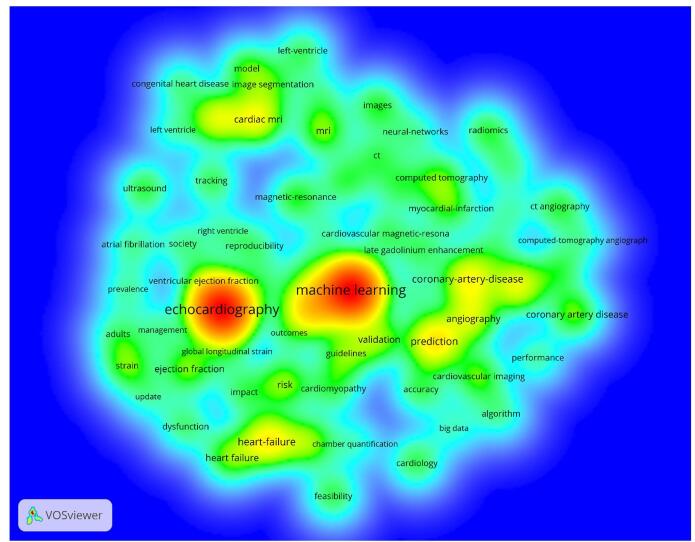
Density visualization map of the keywords. (This figure displays the keywords with the highest frequency (colored in red, orange, and yellow). It highlights the topics that researchers have focused on extensively so far, such as machine learning, echocardiography, and diagnosis.).

The analysis of keywords identified several that frequently occurred within research on AI in cardiovascular imaging. The top ten most prominent keywords included machine learning (*n* = 300), echocardiography (*n* = 298), diagnosis (*n* = 94), prediction (*n* = 79), heart failure (*n* = 71), coronary artery disease (*n* = 64), cardiac CT (*n* = 61), cardiac MRI (*n* = 61), convolutional neural network (*n* = 58), and angiography (*n* = 52) (Fig. 14).

**Figure 15.**
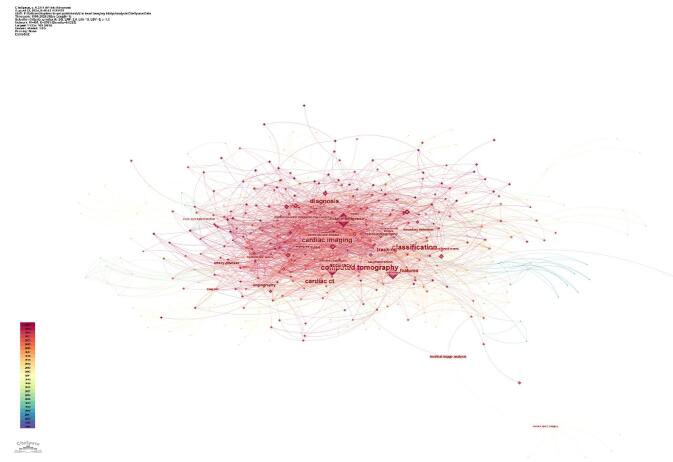
Keywords with the high centrality the field of artificial intelligence in cardiovascular imaging. (This figure presents the keywords with the highest centrality in the global research network on artificial intelligence in cardiovascular imaging. Centrality reflects the importance of these keywords in connecting different research areas. Terms such as classification, computed tomography, cardiac imaging, diagnosis, and cardiac CT appear with high centrality, indicating their crucial role in bridging various topics and studies.).

The analysis of central keywords, which reflected the most influential terms within the research network, revealed the following top ten: classification (0.10), computed tomography (0.10), cardiac imaging (0.10), diagnosis (0.09), cardiac CT (0.09), artificial intelligence (0.07), tracking (0.07), accuracy (0.07), features (0.07), and segmentation (0.06) (Fig. 15).

**Figure 16.**
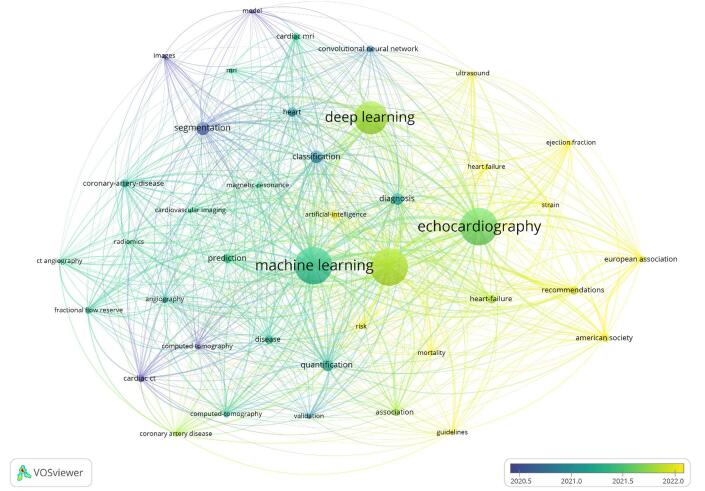
Overlay visualization of keywords in the field of artificial intelligence in cardiovascular imaging. (This figure offers an overlay visualization of keyword trends in research on artificial intelligence in cardiovascular imaging. The overlay color scheme reflects the chronological appearance of keywords, with newer terms in yellow and older ones in blue. The figure shows the evolution of research focus, with earlier topics such as machine learning and diagnosis giving way to more recent areas of interest, including ejection fraction, risk, and heart failure.).

Our analysis also identified the top ten most recent keywords emerging in the field of AI in cardiovascular imaging, reflecting the latest trends and focus areas in research. These keywords included ejection fraction, risk, heart failure, American Society, European Association, artificial intelligence, guidelines, mortality, strain, and recommendations. These terms highlighted current and emerging areas of interest within the research community, indicating where future studies might be concentrated (Fig. 16).

**Figure 17.**
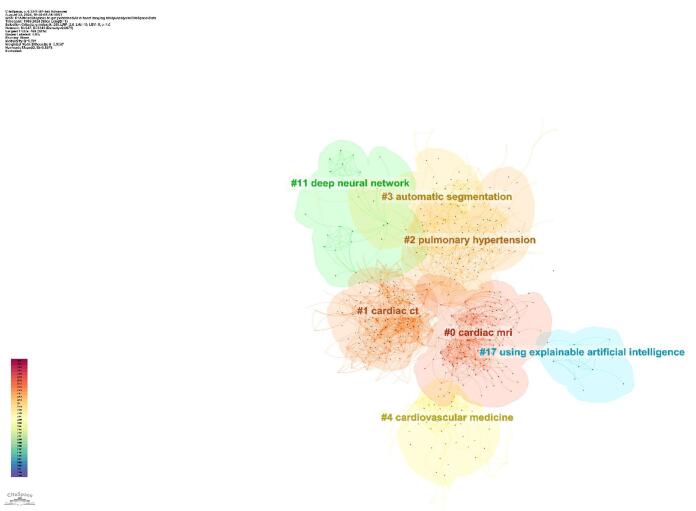
Cluster analysis of the topics in the field of artificial intelligence in cardiovascular imaging. (This figure shows the results of a cluster analysis of research topics in artificial intelligence for cardiovascular imaging. Key clusters identified include cardiac MRI, cardiac CT, pulmonary hypertension, and automatic segmentation, representing central themes in the application of artificial intelligence for cardiovascular diagnostics.).

The cluster analysis identified seven main clusters, representing significant areas of research focus. These clusters included Cardiac MRI (#0), Cardiac CT (#1), Pulmonary Hypertension (#2), Automatic Segmentation (#3), Cardiovascular Medicine (#4), Deep Neural Network (#11), and Using Explainable Artificial Intelligence (#17) (Fig. 17).

**Figure 18.**
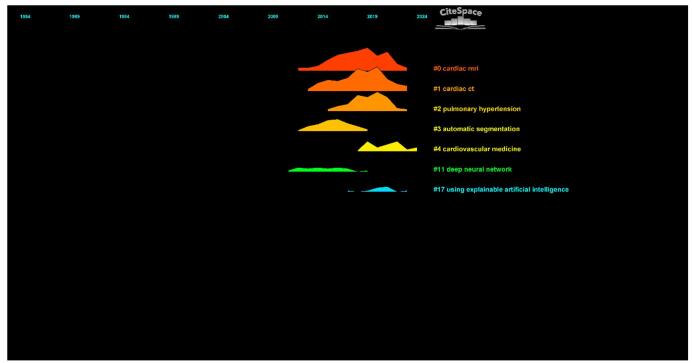
Time trend analysis of topics in the field of artificial intelligence in cardiovascular imaging (This figure presents a time trend analysis of key research topics in artificial intelligence for cardiovascular imaging, illustrating how research priorities have evolved over time. The figure shows that topics such as cardiac MRI, cardiac CT, and pulmonary hypertension have gained significant attention in recent years, while earlier research focused more on automatic segmentation and deep neural networks.).

The time trend analysis illustrated the evolution of research clusters in the field over time. The analysis showed that research priorities had shifted, with certain clusters gaining more focus in recent years. Notably, clusters, such as Cardiac MRI (#0), Cardiac CT (#1), and Pulmonary Hypertension (#2), have received significant attention in the past years (Fig. 18).

### Three-field and thematic analysis

**Figure 19.**
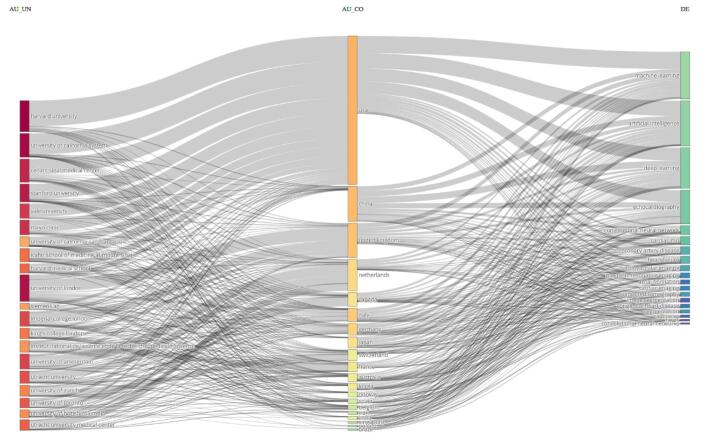
The three-field diagram. (This figure presents the connections between major research institutions, countries, and key research fields, including artificial intelligence and cardiac imaging. This diagram highlights the strong associations between American institutions and areas such as machine learning and artificial intelligence, which emphasize the USA’s dominant role in these fields. Additionally, the diagram highlights international collaborations, particularly in advanced fields like deep learning and convolutional neural networks.).

The three-field diagram illustrated the interconnections between prominent institutions, countries, and research fields, showcasing global contributions to artificial intelligence and cardiac imaging (Fig. 19). On the left, major institutions such as Harvard University, the University of California System, Stanford University, the Mayo Clinic, and Cedars-Sinai Medical Center were highlighted. These institutions were recognized for their leadership in both medicine and computational research, and they were prominently featured in the diagram. The middle section represented the countries of origin for these institutions, with the USA dominating this part due to its large number of research institutions, emphasizing its leading role in the field. Other countries, including the UK, the Netherlands, and China, also contributed significantly, although on a smaller scale compared to the USA.

The right column listed research areas, such as machine learning, artificial intelligence, deep learning, and echocardiography. The diagram clearly showed strong links between these institutions and the key research fields driving innovation in medical technologies. For example, machine learning and artificial intelligence were extensively connected to American institutions, demonstrating the USA’s significant investment and focus on these areas. Additionally, advanced fields like deep learning and convolutional neural networks were closely associated with institutions from various countries, reflecting ongoing collaborative efforts to push these technologies forward.

Specific fields related to cardiovascular health, including echocardiography, cardiac MRI, coronary artery disease, and heart failure, remain heavily linked to medical research centers and universities. This highlights the intersection between computational advancements and their applications in diagnosing and treating heart-related conditions. Furthermore, the diagram shows the significant application of deep learning and convolutional neural networks in medical imaging, segmentation, and diagnostics, emphasizing the dense connections between research institutions and these specialized fields.

## Discussion

Our study’s bibliometric analysis reflected the continuous growth and rapid expansion of AI applications in cardiovascular imaging. We presented an overview of this evolving landscape. The significant increase in publications from 2018 to 2023 demonstrates the growing focus and investment in technologies aimed at enhancing cardiovascular imaging. This trend is driven by the demand for more accurate, efficient, and reproducible diagnostic tools in the management of cardiovascular diseases^[17,59]^.

There has been a growing number of publications, particularly after 2018, indicating that an increasing number of researchers have recognized the potential for AI to revolutionize cardiovascular imaging. The widespread availability and enhanced applicability of deep learning and machine learning methods in addressing complex medical imaging challenges align with this growing trend^[60]^.

AI can enhance clinical workflows as the demand for cardiovascular imaging investigations increases. This is achieved by automating tasks and reducing human error in processes such as image segmentation and interpretation. Echocardiography, CT scans, nuclear imaging, and cardiac magnetic resonance (CMR) are examples of multimodality imaging that currently utilize AI^[61–63]^. For instance, Tan and colleagues developed a novel method for automatically segmenting the left ventricle (LV) using a convolutional neural network (CNN) regression model^[64]^. Their approach incorporated specific physical constraints into the segmentation process by focusing on the radial distances between the LV center point and the endo- and epicardial contours in polar space. When tested against the Left Ventricle Segmentation Challenge (LVSC) dataset, this method achieved the best-published result among automated approaches, with a Jaccard index of 0.77. It was also evaluated in the Kaggle Second Annual Data Science Bowl, where it ranked tenth with a Continuous Ranked Probability Score (CRPS) of 0.0124, the original challenge’s ranking metric. The findings demonstrated that integrating CNN regression with domain-specific characteristics significantly improves clinical segmentation tasks.

There was a significant international presence in AI for cardiovascular imaging, as evidenced by the networks of collaboration and geographical distribution. The United States led in terms of publication output, followed by China and England. The emphasis on the US and UK within the global research network highlights their critical roles in advancing research, promoting international collaborations, and shaping the future of this field. By facilitating the international exchange of information, these partnerships are crucial to the development of AI in cardiovascular imaging. The global and collaborative nature of research in this area was further demonstrated by the major contributions from European countries, particularly England, Italy, and Germany. Leading institutions, such as King’s College London, Harvard University, the Mayo Clinic, and the University of California System, were at the forefront of advancements in AI for cardiovascular imaging^[19,65–67]^. For AI to continue advancing and being clinically translated into cardiovascular care, it is essential that renowned institutions like these continue to train the next generation of researchers and physicians.

The significant contributions of countries like the United States, China, and England in AI-driven cardiovascular imaging research can be attributed to several key factors, including policy support, substantial scientific research investments, and robust international collaboration^[68–70]^. For instance, national policies in the U.S. and China have prioritized AI development in healthcare, fostering an environment conducive to innovation^[71–74]^. Additionally, substantial investments in research and development by governments and private sectors in these countries have facilitated technological advancements^[68,75,76]^. International collaborations, particularly between the U.S. and European countries, have further strengthened research networks, enabling knowledge exchange and shared innovation^[77–79]^. These factors collectively contribute to the high research output observed in these regions, underscoring the importance of policy, funding, and cooperation in shaping research trends.

A broader acknowledgment of international collaboration is essential to provide a more global perspective on the advancements in AI for cardiovascular imaging. While the United States has been a key leader in this field, it is important to recognize the significant contributions from countries beyond the US, such as those in Europe and East Asia. Countries like England, Germany, and the Netherlands in Europe, as well as China and Japan in East Asia, have made substantial contributions to both the volume and quality of research in AI-driven cardiovascular imaging. This global collaboration not only enriches the research landscape but also highlights the interconnected nature of scientific progress in this area. The collaborative efforts between these regions underscore the collective global commitment to improving diagnostic accuracy and patient care through AI, advancing the field in ways that no single region could achieve alone.

A wide variety of scholarly journals, each with its distinct focus, influenced the field of artificial intelligence applications in cardiovascular imaging. Leading journals in this area included Circulation: Cardiovascular Imaging, Journal of Cardiovascular Magnetic Resonance, European Heart Journal – Cardiovascular Imaging, and IEEE Transactions on Medical Imaging, all known for their high standards and significant impact^[53,55,67]^. These journals provided insights into both foundational approaches and new applications of AI in cardiovascular imaging, serving as key platforms for sharing groundbreaking research. Circulation: Cardiovascular Imaging and European Heart Journal – Cardiovascular Imaging were particularly notable for their emphasis on the clinical integration of AI and modern imaging technologies. Essential for both researchers and clinicians, the articles published in these journals explored the diagnostic, prognostic, and therapeutic potential of AI-driven imaging^[51,53,55]^.

By examining the most-cited papers in AI applications in cardiovascular imaging, we observed the significance these investigations had for moving the field forward. In order to improve image quality under difficult situations like low-dose settings, the most cited publication, “Generative adversarial networks for noise reduction in low-dose CT,” published in 2017 and with 664 citations, established a standard for incorporating AI approaches into imaging procedures^[51]^. This study investigated the use of Generative Adversarial Networks (GANs) to reduce noise in low-dose CT images by transforming them into routine-dose quality images. A CNN was trained as a generator to minimize voxel wise loss, while an adversarial CNN was trained as a discriminator to differentiate between generated and routine-dose images. Experiments on both an anthropomorphic phantom and patient cardiac CT images demonstrated that the CNN trained with only voxelwise loss achieved the highest peak signal-to-noise ratio. However, CNNs trained with adversarial loss better replicated the appearance of routine-dose images, leading to improved noise reduction and accurate coronary calcium scoring. The approach proved feasible, with processing times under 10 seconds per CT volume, indicating its potential for clinical application in enhancing low-dose CT imaging quality. Also, the 2018 paper with 466 citations titled “Fully automated echocardiogram interpretation in clinical practice: feasibility and diagnostic accuracy” followed it closely^[46]^. The study explored the feasibility and diagnostic accuracy of a fully automated pipeline for echocardiogram interpretation, leveraging advances in computer vision. Using a dataset of over 14 000 echocardiograms collected over a decade, the researchers trained convolutional neural networks to automate key tasks: view identification, cardiac chamber segmentation, quantification of cardiac structure and function, and disease detection. The CNNs achieved high accuracy in identifying echocardiographic views, with 96% accuracy for the parasternal long axis, and demonstrated strong agreement with manual measurements for chamber volumes and ejection fraction. The automated system also successfully detected hypertrophic cardiomyopathy, cardiac amyloidosis, and pulmonary arterial hypertension with C statistics of 0.93, 0.87, and 0.85, respectively. These results suggested that automated echocardiogram interpretation could democratize access to cardiac evaluation, particularly in primary care and rural settings, while also supporting large-scale research through standardized analysis of archived echocardiograms.

The 2019 paper titled “Clinical applications of machine learning in cardiovascular disease and its relevance to cardiac imaging” by Al’Aref, *et al* explored key areas where machine learning (ML) had been integrated into cardiovascular disease management, especially within cardiac imaging^[55]^. The paper covered various applications, including echocardiography, electrocardiography (ECG), coronary computed tomography angiography (CCTA), and myocardial perfusion imaging. For example, in echocardiography, ML had been used to analyze left ventricular ejection fraction and longitudinal strain, with a mean bias of −0.3 and a 95% confidence interval ranging from −1.5 to 0.9, resulting in an intraclass correlation coefficient of 0.83 compared to manual tracing.

The 2016 paper titled “A combined deep-learning and deformable-model approach to fully automatic segmentation of the left ventricle in cardiac MRI” by Avendi, *et al* introduced an advanced technique for automatically segmenting the left ventricle in cardiac MRI^[52]^. This hybrid approach combined deep learning with a deformable model to achieve precise segmentation, which was crucial for evaluating cardiac function. The deep learning component utilized CNNs to extract relevant features from MRI images, while the deformable model fine-tuned the segmentation by using prior shape information of the left ventricle. The study showed that this integrated method significantly enhanced segmentation accuracy compared to traditional techniques, achieving a Dice similarity coefficient (DSC) of 0.93 when compared to manual annotations. Additionally, the algorithm demonstrated robustness across various MRI datasets and outperformed other state-of-the-art methods in both accuracy and speed. The deep learning model first identified the region of interest, followed by the deformable model, which refined the contours to ensure high precision. This approach reduced the need for manual intervention, streamlined the workflow in clinical settings, and provided reliable quantification of cardiac parameters essential for diagnosing heart diseases. These advancements highlighted the potential for deep learning and deformable models to automate complex tasks in cardiac imaging, ultimately improving clinical decision-making.

By analyzing citation bursts, influential studies that gained attention in a short period of time in the field of AI and cardiovascular imaging were identified. One of these papers was the 2012 work titled “Imagenet classification with deep convolutional neural networks” by Krizhevsky, *et al* ^[80]^. This groundbreaking study introduced a novel approach to object classification in large image datasets. The authors presented a deep learning model based on CNNs that significantly outperformed traditional methods in image classification tasks, particularly in the ImageNet Large-Scale Visual Recognition Challenge (ILSVRC). The deep network, consisting of five convolutional layers and three fully connected layers, utilized ReLU (Rectified Linear Unit) activations to accelerate training and dropout for regularization to prevent overfitting. Notably, the model achieved a top-5 error rate of 17.0% in the ILSVRC-2010 competition, a substantial improvement over previous state-of-the-art model. A key innovation was the use of GPU-accelerated training and large datasets, enabling the model to effectively handle over a million high-resolution images and 1000 object categories. This paper paved the way for the widespread adoption of deep CNNs in computer vision, influencing subsequent advancements in image recognition and classification across various domains.

The 2015 paper titled “Fully automated versus standard tracking of left ventricular ejection fraction and longitudinal strain” by Knackstedt, *et al*^[4]^ introduced a machine learning-enabled software for the automatic measurement of left ventricular (LV) function in echocardiography^[81]^. This fully automated method aimed to address the subjectivity and variability associated with manual and visual assessments of LV ejection fraction (EF) and longitudinal strain (LS). The software, AutoLV, applied a machine learning algorithm to rapidly analyze apical 4- and 2-chamber views from echocardiographic images. It provided fully automated LV volumes and EF within seconds, reducing the need for manual tracing, which can be time-consuming and inconsistent. The method demonstrated strong agreement with manual EF measurements, yielding intraclass correlation coefficients (ICC) of up to 0.83, and performed robustly across multiple data sets with an average analysis time of only 8 seconds per patient. The paper highlighted the potential of automation to standardize EF and LS measurements, reducing variability between operators and centers, and providing accurate, reproducible cardiac function assessments. This advancement is poised to enhance efficiency in clinical workflows and improve patient care by offering reliable cardiac parameters without manual intervention.

By analyzing keywords, we gained insight into which areas of AI in cardiovascular imaging received the most attention and funding. The most frequently used keywords, such as machine learning, echocardiography, and diagnosis, indicated the research’s main focus, emphasizing the use of AI methods to improve diagnostic precision and efficiency. Terms like prediction, heart failure, and coronary artery disease highlighted specific medical applications of AI that saw significant progress, particularly in the prognosis and management of complex cardiovascular diseases^[17,19]^. By analyzing these important keywords, we gained a clearer understanding of the fundamental concepts behind the studies. Words such as classification, computed tomography, and cardiac imaging stood out as key components of the research framework. These terms revealed both the primary areas of interest and the most commonly used approaches and tools in this field. Essential tasks in diagnostic imaging, like classification and segmentation, attracted considerable attention from AI researchers due to the technical focus on developing models that could accurately identify and segment cardiac images.

Cluster analysis showed that different groups of researchers concentrated on various topics. Clusters such as Pulmonary Hypertension (#2) and Automatic Segmentation (#3) reflected specialized uses of AI in addressing specific cardiovascular conditions and imaging challenges, while Cardiac MRI (#0) and Cardiac CT (#1) highlighted ongoing efforts to improve imaging modalities through AI. The identification of clusters like Deep Neural Networks (#11) and Explainable Artificial Intelligence (#17) suggested that researchers were increasingly focused on developing AI models that were both powerful and easy to understand and apply in clinical settings. By analyzing the time history of these clusters, we observed that research objectives had shifted over the years, with a current emphasis on fields like cardiac MRI and computed CT^[19,46,53,55,59,65,67,82]^, where AI had driven significant breakthroughs in imaging techniques. This development indicated the field’s growth, as researchers built on earlier work to explore more advanced and clinically applicable uses of AI in cardiac imaging.

Advancements in AI are increasingly being integrated into cardiac imaging, enhancing diagnostic accuracy and efficiency in clinical practice. These technologies are transitioning from theoretical concepts to practical tools that significantly improve patient care^[17,32,83]^. In echocardiography, AI algorithms facilitate the automated assessment of cardiac function, including systolic and diastolic performance. This automation accelerates evaluations and enhances reproducibility, aiding clinicians in diagnosing conditions such as heart failure^[84,85]^. Similarly, in CMR, AI streamlines image acquisition and analysis. It assists in tasks like image segmentation and interpretation, improving workflow efficiency and diagnostic precision^[86,87]^. In coronary CT angiography (CCTA), AI aids in detecting significant coronary artery disease. It assists in identifying coronary atherosclerosis, supporting clinicians in making informed decisions regarding patient management^[88,89]^. AI also plays a crucial role in electrocardiogram (ECG) analysis. Algorithms analyze ECG data to detect conditions such as atrial fibrillation and other arrhythmias. This early detection facilitates timely intervention, potentially improving patient outcomes^[90,91]^. Moreover, AI enhances the efficiency of cardiac imaging workflows by automating routine tasks, reducing interpretation times, and minimizing human error. This optimization allows clinicians to focus more on patient care^[19,92]^. These integrations demonstrate AI’s tangible impact on cardiac imaging, moving beyond theoretical potential to practical, real-world applications that enhance patient care^[92,93]^.

In the field of cardiovascular imaging, the use of AI holds incredible promise but also raises several important concerns. One of the biggest challenges is the “black box” nature of many AI systems, particularly deep learning models, where the decision-making process is not always clear^[94-97]^. This lack of transparency makes clinicians hesitant to rely solely on AI-driven diagnoses or predictions, especially in critical, life-threatening situations. Without understanding how these systems reach their conclusions, it is difficult for healthcare providers to place full trust in them when patient lives are at stake^[55,98-101]^.

Another significant issue is data privacy and security. AI systems require large amounts of patient data for training and validation, which raises concerns about the potential exposure or misuse of sensitive health information^[102,103]^. Additionally, there is a risk that AI models could unintentionally embed biases from the data they are trained on, potentially leading to unequal care. For instance, if a system is developed using data primarily from one demographic group, it may not perform as well for others, potentially worsening existing health disparities^[104,105]^.

Regulatory and ethical questions also need to be addressed. The rapid advancement of AI technologies is outpacing the ability of current regulations to keep up^[105,106]^. A key concern is accountability^[107,108]^. When AI-based decisions result in poor patient outcomes, it is unclear who should be held responsible. This is particularly critical in cardiovascular imaging, where accuracy is paramount and errors can have severe consequences^[109,110]^.

## Limitation

Although our bibliometric study covers a wide scope, there are some limitations to consider. First, our analysis only included articles from the Web of Science Core Collection, and we prioritized it because it is widely regarded for its rigorous indexing standards, comprehensive coverage of high-impact journals, and robust citation data, which are critical for performing reliable citation-based analyses. Compared to Scopus and PubMed, Web of Science offers more sophisticated citation tracking tools, enabling deeper exploration of citation networks and collaboration patterns. Although Web of Science is a reputable source for high-quality academic work, it does not cover all relevant literature. Studies from other databases like Scopus and PubMed were not part of the analysis, which may have led to the exclusion of some contributions. Furthermore, non-indexed journals, which might feature regionally important research, were also omitted. This could result in a bias in our findings, as excluding studies from certain databases might lead to an underrepresentation of key regions or alternative methods in artificial intelligence applications in cardiovascular imaging research.

Secondly, our study was restricted to articles published in English, which may have led to the exclusion of important research available in other languages. This language limitation could especially impact the representation of non-English-speaking regions involved in cardiovascular research, such as areas of Asia, Latin America, and Europe. Incorporating non-English studies in future bibliometric analyses would enhance the global perspective and highlight diverse scientific contributions.

Third, the bibliometric tools employed in this study – VOSviewer, CiteSpace, and Biblioshiny – depend on citation data. Although these tools are valuable for identifying research trends, it is important to recognize that citation counts do not always provide a precise measure of research quality. Factors such as the prominence of particular journals, collaborations among authors, or the popularity of certain research topics can influence citation counts, leading to a potentially biased or incomplete depiction of scientific impact. In addition to these factors, citation counts are influenced by regional disparities in research funding and institutional visibility, which can skew the representation of research output from different regions. For instance, well-funded regions such as the US and UK often dominate citation-based metrics, not necessarily because their research is of superior quality, but due to better infrastructure and higher visibility. Citation metrics can also be biased toward studies published in high-impact journals, which tend to attract more attention and citations, leaving relevant research published in lower-tier journals underrepresented. Furthermore, citation counts do not account for the “time lag” between publication and citation accumulation. Newer studies, despite their potential relevance and quality, may not yet have gained significant citation traction, whereas older studies that have had more time to accumulate citations may disproportionately influence citation-based metrics. Additionally, self-citations or citation cartels can artificially inflate citation counts, which further distorts the true impact of a study. As a result, highly relevant studies published in less well-known journals or those from emerging research hubs may not be fully represented in this analysis, which limits the ability to capture the full diversity and breadth of research in this rapidly evolving field.

Additionally, the study is based on publications up to August 2024. Given the rapid advancements in AI technologies within cardiovascular imaging, it is important to acknowledge that the field is continuously evolving. As a result, this analysis may not fully reflect the most recent developments and innovations in AI applications, highlighting the need for future updates to capture the latest trends and breakthroughs. Finally, the bibliometric approach may be influenced by publication bias, with a predominance of positive or novel findings potentially coloring the results. The variability in study methodologies and publication practices across different journals and regions may also affect the comparability and generalizability of the findings.

Lastly, AI in cardiovascular field is rapidly advancing. As new studies emerge, especially with technological developments such as machine learning, genomics, and personalized medicine, the trends observed in our bibliometric analysis may evolve quickly. It is crucial to recognize that our study offers a snapshot of the field as of 2024, and regular updates will be needed to capture future advancements in the literature.

## Conclusion

Based on the results of our bibliometric analysis, we draw the following conclusions: Research output related AI applications in cardiovascular imaging has shown significant growth over the years, reflecting a rising global interest in leveraging AI technologies to enhance diagnostic and therapeutic strategies. The USA has emerged as a major contributor and central hub for international collaboration in this field, with numerous influential studies and high-impact publications driving the discourse. Key journals, such as “Frontiers in Cardiovascular Medicine” and “International Journal of Cardiovascular Imaging,” have been pivotal in disseminating groundbreaking research, underscoring their importance in academic communication and the propagation of new knowledge. Current research hotspots are centered around the development and application of advanced machine learning and deep learning algorithms, particularly convolutional neural networks, for improving the accuracy and efficiency of cardiovascular imaging. Emerging trends also focus on automated quantification, real-time risk stratification, and the integration of AI with existing clinical workflows to enhance personalized care. In order to facilitate the widespread adoption of AI technology in healthcare systems, future research should focus on resolving the difficulties associated with clinical integration, such as regulatory permission, data protection, and ethical considerations. To make sure AI-driven discoveries are useful and significant for routine clinical usage, cooperation between researchers, physicians, and policymakers will be crucial.

## Data Availability

The data supporting the findings of this study were obtained from the Web of Science Core Collection database. These data can be made available upon reasonable request to the corresponding author.
